# Inverted Expression Profiles of Sex-Biased Genes in Response to Toxicant Perturbations and Diseases

**DOI:** 10.1371/journal.pone.0056668

**Published:** 2013-02-14

**Authors:** Choong Yong Ung, Siew Hong Lam, Xun Zhang, Hu Li, Louxin Zhang, Baowen Li, Zhiyuan Gong

**Affiliations:** 1 Department of Biological Sciences, National University of Singapore, Kent Ridge, Singapore; 2 Bioinformatics Programme, Institute of Biological Sciences, University of Malaya, Kuala Lumpur, Malaysia; 3 Graduate School for Integrative Sciences and Engineering, National University of Singapore, Kent Ridge, Singapore; 4 Department of Physics and Centre for Computational Science and Engineering, National University of Singapore, Kent Ridge, Singapore; 5 Department of Biomedical Engineering, Boston University, Boston, Massachusetts, United States of America; 6 Department of Mathematics, National University of Singapore, Kent Ridge, Singapore; Auburn University, United States of America

## Abstract

The influence of sex factor is widely recognized in various diseases, but its molecular basis, particularly how sex-biased genes, those with sexually dimorphic expression, behave in response to toxico-pathological changes is poorly understood. In this study, zebrafish toxicogenomic data and transcriptomic data from human pathological studies were analysed for the responses of male- and female-biased genes. Our analyses revealed obvious inverted expression profiles of sex-biased genes, where affected males tended to up-regulate genes of female-biased expression and down-regulate genes of male-biased expression, and vice versa in affected females, in a broad range of toxico-pathological conditions. Intriguingly, the extent of these inverted profiles correlated well to the susceptibility or severity of a given toxico-pathological state, suggesting that inverted expression profiles of sex-biased genes observed in this study can be used as important indicators to assess biological disorders.

## Introduction

In spite of the ubiquity of sexual reproduction in multicellular eukaryotes, it is considered to be evolutionary expensive as males do not reproduce by themselves, leading to “cost of males” in ecology and evolution [1], [2]. However, sexual reproduction has the advantage of producing progenies that are capable of adapting to dynamic environments [3]–[5]. Sexual recombination is thought to act as an important mechanism in releasing mutation meltdown caused by mutation accumulation [6] as well as to act as an adaptation mode to resist invasion of parasites [7]–[9].

Since sex factor plays an important role in environmental adaptation, where diseases can act as a selective pressure in evolution [10]–[12], it is not surprising that a number of diseases are sexually dimorphic in prevalence. Examples are tuberculosis [13], hepatitis C virus infection [14], schizophrenia [15], rheumatoid arthritis [16], coronary artery calcification [17] and ischemic heart disease [18], with many of them shows higher prevalence in males. Although the association of sex factor to many diseases is “common sense”, the actual contribution of sex factor to the etiology of diseases remains unresolved. Furthermore, despite well recognized differences between males and females in their pharmacokinetics and pharmacodynamics in response to drugs [19], sex factor is rarely considered in the whole drug discovery pipeline [20]. Women are generally underrepresented in biomedical researches [21], [22]; consequently, women experienced 1.5 times higher risk than men in developing adverse drug reactions [23]. Thus, it is important to investigate the importance of sex factor in both basic and biomedical research.

Differences between males and females in both morphology and physiology have been well appreciated as sexual dimorphic traits. The advent of genome-wide microarray analyses further indicated that sexual dimorphism can also occur at the gene expression level. Now increasing evidence indicates that sex-biased gene expression is not just limited to gonads, as tens to thousands of genes showed differential expression between the two sexes in numerous non-gonadal tissues examined, such as liver [24], [25], kidney [26], lacrimal gland [27], brain [28], [29], adipose tissue [30], etc. Hence, the phenomenon of sex-biased gene expression is in fact quite common and many of these sex-biased expressed genes are also tissue-specific genes [30].

Here, we defined sex-biased genes as genes with sexual dimorphic expression, with male-biased genes as those predominantly expressed in males and vice versa for female-biased genes. The term “sex-biased genes” will be used in referring to both male-biased and female-biased genes hereafter. To investigate sex-dependency to various toxico-pathological and disease conditions, we used the zebrafish as a model to study sex-dependent response to toxicants. In addition, we also retrieved transcriptome data from Gene Expression Omnibus for the association of sex to human diseases. Our analyses revealed striking observation that sex-biased genes in both zebrafish and human exhibited generally similar expression behavior in response to toxicological perturbations (zebrafish) and pathological conditions (human). Both fish and human show inverted expression profiles of sex-biased genes, where affected males tended to up-regulate female-biased genes and down-regulate male-biased genes, and vice versa in affected females. Intriguingly, the extent of these inverted profiles also correlated well with the severity or susceptibility to a given toxico-pathological state, suggesting the importance of sex-biased genes in playing active roles in regulating normal physiological functions.

## Methods

### Transcriptomic data

Transcriptomic data used in this work, together with their respective Gene Expression Omnibus (GEO) series accession, are summarized in Table 1. Zebrafish toxicogenomic data were generated from our laboratory by using a DNA microarray platform as described in previous publications [31]–[34]. In these studies, zebrafish were treated with cadmium (II), arsenic (V), chloroaniline (CA), and p-nitrophenol (NP). These chemicals were chosen because they serve as representatives of selected environmental toxicants that are potential health hazards to various organisms including humans, hence having considerable public health concern [35]. Relevant human transcriptome data were collected from GEO and their respective series accession are also given in Table 1. Due to limited microarray experiments performed to address sex-dependency on the lack of clear indication on gender for many data sets, we identified only 9 published microarray series from GEO [36]–[44], covering 9 different human tissues and 17 distinct pathological cases (Table 1), suitable for the current study.

**Table 1:** Summary of microarray data used in this work.

GEO Series Accession	Data Description	Organism	Organ/Tissue	Reference
GSE41623	Cd; Liver treated with Cadmium (II) chloride, 30 µg from 8 to 96 hr	Zebrafish (Male)	Liver tissues	Unpublished
GSE41622	Cd; Liver treated with Cadmium (II) chloride, 30 µg from 8 to 96 hr	Zebrafish (Female)	Liver tissues	Unpublished
GSE3048	As; Liver treated with Arsenic (V), 15 ppm (∼192 µM) from 8 to 96 hr	Zebrafish (Male)	Liver tissues	31
GSE30062	As; Liver treated with Arsenic (V), 15 ppm (∼192 µM) from 8 to 96 hr	Zebrafish (Female)	Liver tissues	32
GSE30055	CA; Liver treated with Chlroroaniline 20 mg/L from 8 to 96 hr	Zebrafish (Male)	Liver tissues	32
GSE30057	CA; Liver treated with Chlroroaniline 20 mg/L from 8 to 96 hr	Zebrafish (Female)	Liver tissues	32
GSE30058	NP; Liver treated with Nitrophenol, 7 mg/L from 8 to 96 hr	Zebrafish (Male)	Liver tissues	32
GSE30060	NP; Liver treated with Nitrophenol, 7 mg/L from 8 to 96 hr	Zebrafish (Female)	Liver tissues	32
GSE3467	Papillary Thyroid Carcinoma	Human	Thyroid tissues	36
GSE4107	Colorectal Cancer	Human	Colonic mucosa	37
GSE5081	Gastric Helicobacter pylori Infection	Human	Gastric biopsy	38
GSE7621	Parkinson's Disease	Human	Substantia nigra tissue	39
GSE10135	Airway Epithelial Cells from Smoker	Human	Airway epithelial cells	40
GSE10927	Adenoma and Adrenocortical Carcinoma	Human	Adrenal cortex	41
GSE11348	Rhinovirus Infection	Human	Nasal srcapings	42
GSE11882	Aging in Entorhinal Cortex, Hippocampus, Postcentral gyrus, and Superior Frontal gyrus	Human	Postmortem brain tissue	43
GSE13501	Enthesitis-Related Arthritis, Oligoarthritis, Polyarthritis, and Systemic	Human	Peripheral blood tissues	44

### Identification of sex-biased genes

**Figure 1:**
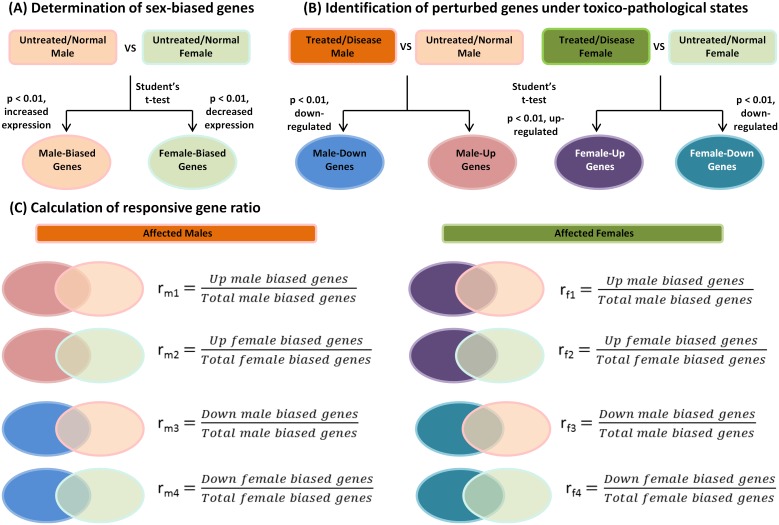
Calculation of responsive gene ratio. (A) Determination of sex-biased genes. Untreated or normal males and females from control groups were compared for sex-dimorphically expressed genes based on p-value<0.01 from Student's t-test with genes expressed at increased levels as male-biased genes and genes expressed at decreased levels as female-biased genes. (B) Identification of perturbed genes under toxico-pathological states. To assess genes that were affected under a given deregulated state, arrays of chemical treated or disease states of the same sex (i.e. male treated/diseased vs. male controls and similar approach for female samples) were compared using Student's t-test. Genes showing p-value<0.01 with increased and decreased expressed transcripts were considered as up- and down-regulated, respectively. (C) Calculation of ratio of sex-biased gene. Genes within the set of sex-biased genes (e.g. male-biased genes) was overlapped with genes from perturbed gene sets to determine proportion of sex-biased genes that were significantly perturbed under a toxico-pathological state. Ratios for responsive male-biased and female-biased genes are calculated by counting the number of affected sex-biased genes (up- and down-regulated) with respect to total male- or female-biased genes (see **METHODS**) and was compiled into Figure 2 and 3.

From the view point of sex-dependent activity of genes, all the expressed genes within an individual can be categorized as either sex-biased or non-sex-biased, with genes showing differential expression or no difference in expression between two sexes. Sex-biased genes can be further categorized into male-biased or female-biased genes, whose expression was significantly biased in males and in females, respectively. For each transcriptomic experiment, controls are referred to untreated healthy individuals. For a respective GEO series, arrays of control males were compared against arrays of control females. Student's t-test was used to access statistical significance for genes that were differentially expressed in males or females where Student's t distribution follow a normal distribution was assumed for expression to the rest of other genes. Genes showing p-value<0.01 with increased expression levels were defined as male-biased genes. Similarly, genes with p-value<0.01 with decreased expression levels were defined as female-biased genes (Figure 1A). Both zebrafish and human data were similarly processed to obtain their respective sex-biased genes.

### Identification of toxicant or disease responsive genes

Before assessing how sex-biased genes behaved under different biological perturbations, it is necessary to identify responsive genes for each sex under these conditions. To determine genes that were affected under a toxicological or a disease state, arrays from the same sex of the treated or diseased states were compared against their respective control group. For instance, arrays of As(V)-treated male zebrafish were compared against untreated male fish in the same experiment to identify genes that were deregulated by As(V) in males. Female data were similarly processed, i.e., treated female fish were compared against untreated female fish. The same procedure was also applied for human data. Genes showing p-value<0.01 from the Student's t-test were considered significantly affected, with those expressed at higher or lower amounts as up- and down-regulated genes, respectively (Figure 1B).

### Assessment of sex dependency in toxico-pathological conditions

**Figure 2:**
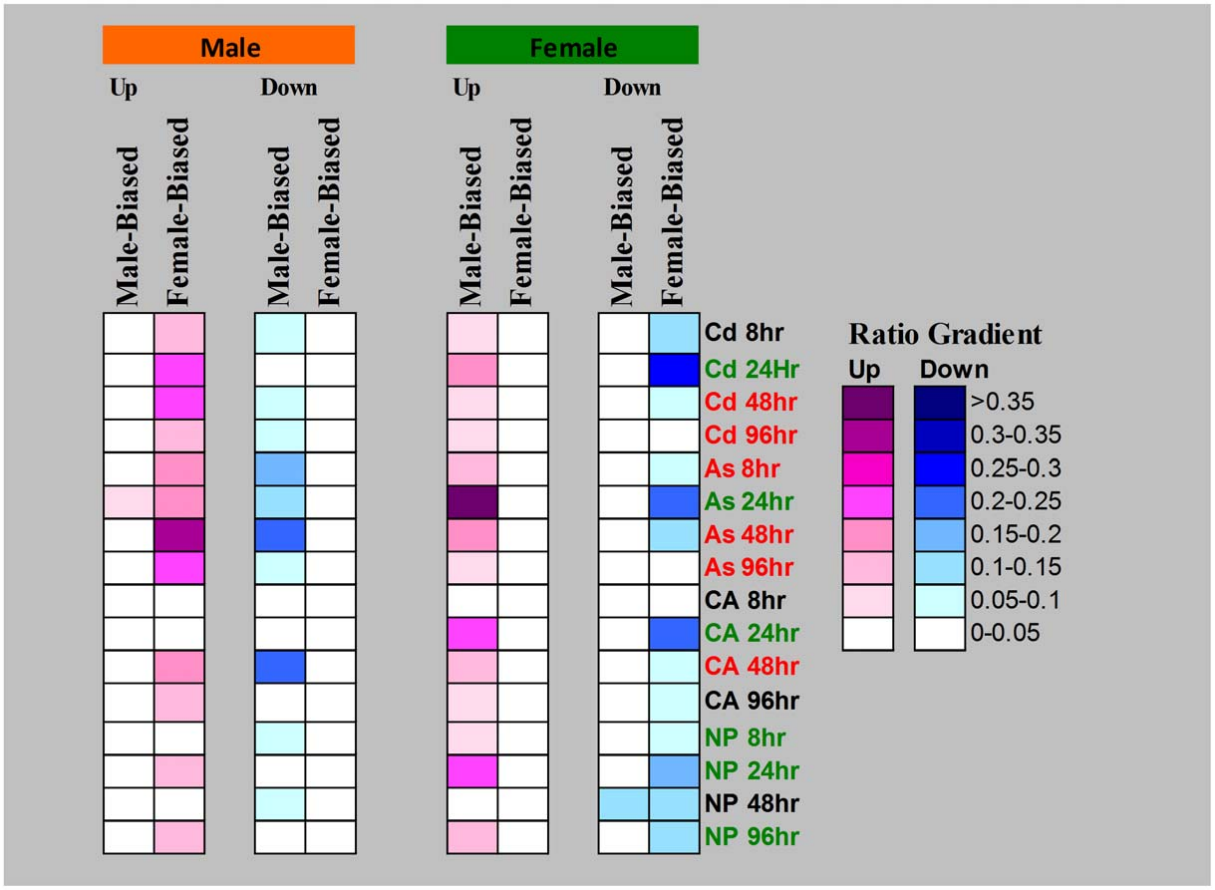
Inverted sex-biased expression profiles of the zebrafish in response to various chemical perturbations. Sex-biased genes from each experiment were determined from males vs. females of physiological states with p-value<0.01 using Student's t-test (Figure 1A). Ratios of responsive sex-biased gene were used to represent the overall responsiveness of male-biased and female-biased genes. Red treatment labels are males showing obvious inverted expression profiles than females. Green treatment labels are females showing obvious inverted expression profiles than males. Black treatment labels are those without clear inverted expression profiles. Abbreviations: Cd, cadmium (II); As, arsenic (V); CA, chloroaniline; NP, p-nitrophenol.

**Figure 3:**
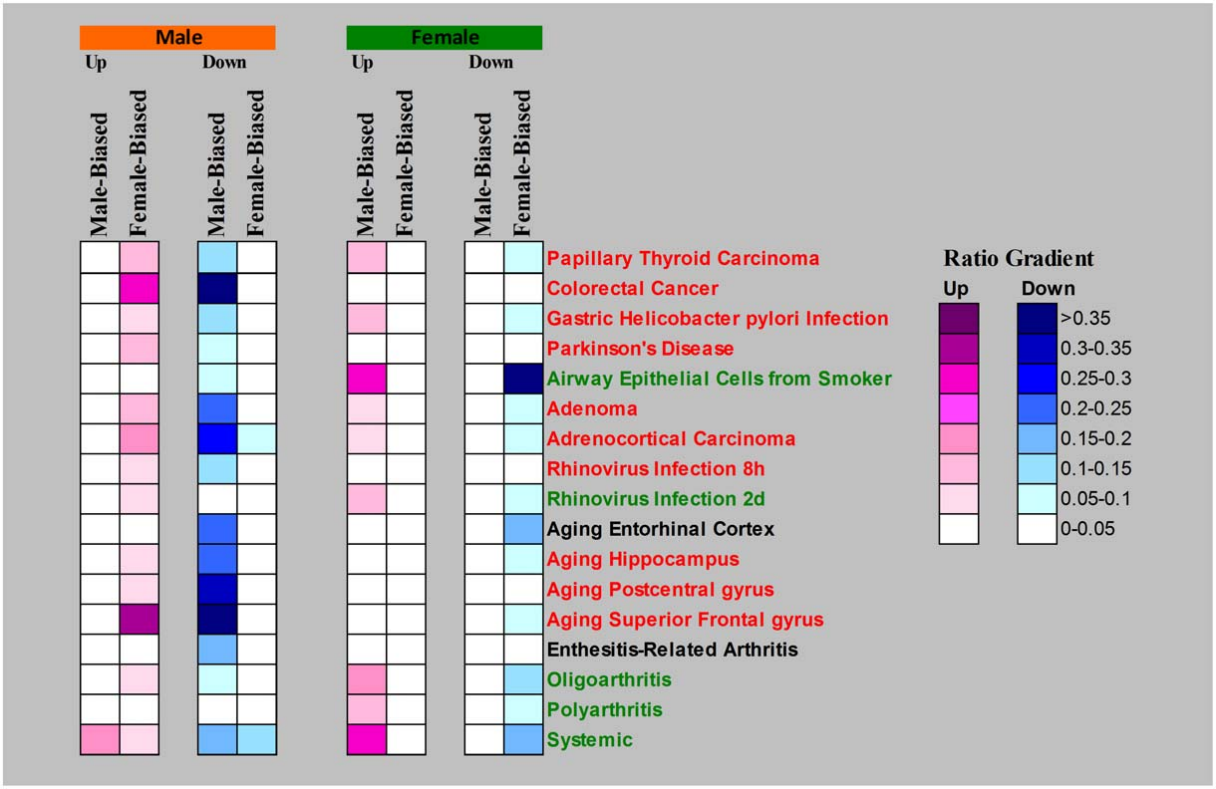
Inverted sex-biased gene expression profiles in human tissues of various pathological states. The description of transcriptomic data used is provided in Table 1. The details of analytical procedure and data representations are described in Figure 2.

To assess sex dependency in different toxico-pathological conditions, several categories of ratios capturing responsiveness of sex-biased genes were calculated as summarized in Figure 1C. Both up- and down-regulated male (or female)-biased genes were calculated for their proportion in total number of male (or female)-biased genes for each toxicological or disease condition. For instance, for a disease state in male, its male-biased gene ratios that were up- and down-regulated were calculated as (Number of up-regulated male-biased genes)/(Total male-biased genes) and (Number of down-regulated male-biased genes)/(Total male-biased genes), respectively. Similar approach was applied to females to calculate ratios of responsive sex-biased genes for both male- and female-biased genes under each experimental condition (Figure 1C). The calculated ratios of responsive sex-biased genes for each condition were compiled giving rise to Figure 2 and Figure 3 for fish and human data, respectively.

### Pathway enrichment for the zebrafish sex-biased genes using WebGestalt

Pathway enrichment for the zebrafish sex-biased genes (Table S1) was performed using WebGestalt, a “WEB-based GEne SeT AnaLysis Toolkit” (http://bioinfo.vanderbilt.edu/webgestalt/). Gene symbols matched to human genes were used for enrichment analysis on KEGG pathway. Hypergeometric test was used as a statistical method for enrichment analysis. Bonferroni method was then used as multiple test adjustment method. Pathways showing adjusted p-value<0.01 with minimum 2 genes in a pathway category were considered as statistical significant.

## Results and Discussion

### Inverted expression profiles of sex-biased genes are widely observed in both fish and human

In this study, we aimed to understand how sex-biased genes behave under a broad spectrum of toxico-pathological conditions. As shown in Figure 1A, sex-biased genes were first defined by comparison of gene expression between male and female control samples from various biological sources. Then, differentially expressed or responsive genes in each toxico-pathological condition were determined (Figure 1B). Finally the ratios of both up- and down-regulated sex-biased genes to the total sex-biased genes were determined (Figure 1C) for evaluation of sex dependence of each toxico-pathological state and the results are presented in Figure 2 for the zebrafish data and in Figure 3 for human data. As shown in these two figures, there were very strikingly similar inverted expression profiles of sex-biased genes across essentially all toxico-pathological conditions for zebrafish and human, i.e., affected males tended to up-regulate female-biased genes and down-regulate male-biased genes. The opposite scenario also applied to affected females. Results in both Figures 2 and 3 indicated that inverted expression of sex-biased expressed genes is a ubiquitous phenomenon but this observation has not been reported thus far.

### The intensity of contrast for an inverted expression profile suggests association to severity or susceptibility for a given toxico-pathological state

Our previous toxicological experiments using zebrafish suggested that there is a general time-dependent response [31], [32], [35]. We wondered whether those responses are correlated with the inverted expression profiles of sex-biased expressed gene. As shown in Figure 2, indeed, fish exposed for longer exposure duration generally had higher contrast (i.e. higher values of responsive ratio of sex-biased genes) of inverted expression of sex-biased genes than those from shorter exposure (Figure 2). However, fish treated for 96 hr generally had lower responsive ratios than those from 24 or 48 hr, which may be related to increased apoptosis and liver damage in 96 hr samples [31], [32], [35]. Red and green sample labels shown in Figure 2 indicate cases where male and female fish show greater ratios for inverted expression profiles, respectively. For cases where male fish show greater inverted expression profiles (red labels in Figure 2), toxicant-treated male fish tended to show higher contrasts for up-regulated female-biased genes than toxicant-treated females for up-regulated male-biased genes. Vice verse is for cases where female fish showed greater inverted expression profiles (green labels in Figure 2). Sex-biased genes for the same sex (e.g. male-biased genes in males) tended to be down-regulated upon chemical treatments albeit affected female fish generally showed higher down-regulation ratios for female-biased genes than down-regulated male-biased genes in affected males.

Likewise, in human pathological conditions, the contrasts of these inverted sex-biased gene expression profiles were correlated to the gender susceptibility of a given pathological condition, with higher susceptibility of males for many of these deleterious states, including Parkinson's disease, and colorectal cancers (red sample labels in Figure 3) [45]. This is consistent with reported studies and clinical observations where males have higher risk in Parkinson's disease and colorectal cancer than females [45]–[47]. Other pathological instances such as adenoma, adrenocortical carcinoma, as well as aging in several brain areas (hippocampus, postcentral gyrus, superior frontal gyrus) also showed obvious inverted expression profiles in males, implicating that males are more susceptible to these pathological complications [43].

However, there are a number of pathological instances where females displayed higher contrast of inverted expression profiles (green sample labels in Figure 3) but they generally showed lesser ratio (contrast) for up-regulated male-biased genes than incidents where males exhibit greater inverted expression profiles (red sample labels) for ratio of up-regulated female-biased genes. This observation is similar to those observed in the zebrafish (Figure 2). However, there are incidents where females suffer higher risk. For instance, female smokers are at higher risk for lung cancer than male smokers [48]. Intriguingly, this was also reflected in Figure 3 (Airway Epithelial Cells from Smoker) with female smokers having very obvious inverted expression profiles for sex-biased genes.

In general, both zebrafish and human data suggested association of inverted expression profiles of sex-biased expressed genes to severity or susceptibility to a given toxico-pathological instance. However, direct evidence and molecular mechanism on these associations to disease susceptibility and severity remain to be investigated.

### Sex-biased genes in the zebrafish liver implied distinct molecular pathways and behavior in inverted expression between males and females

Using statistical criteria applied in this study (Figure 1), we obtained 150 male-biased and 193 female-biased genes in the zebrafish liver (Table S1). Genes that were mapped to human homologs were submitted to WebGestalt for functional enrichment analysis. Results shown in Table 2 suggested distinct molecular pathways that were enriched for male and female fish. Male-biased functional pathways included ubiquitin-mediated proteolysis, base excision repair, pyruvate metabolism and fatty acid biosynthesis. These processes were mainly involved in protein quality control, repair and energetic metabolism via propanoate and fatty acid metabolism. In contrast, focal adhesion as well as several pathways related to lipid metabolisms were enriched in female-biased processes. These included fatty acid beta-oxidation, nuclear receptors in lipid metabolism and toxicity, metabolism of lipids and lipoproteins. Additional processes such as integration of network metabolism and FOXA transcription network were also enriched in female. The enriched sex-biased pathways are shown in Table 2 suggested there were distinct regulations between male and female in their overall hepatic functions, hence emphasizing again the importance of considering gender factor in using animal models for biomedical studies.

**Table 2:** Enriched pathways for sex-biased genes in the zebrafish liver.

Pathway	Genes	Significance Level	Associated Sex
Ubiquitin mediated proteolysis	*BIRC6* (baculoviral IAP repeat-containing 6); *CDC34* (cell division cycle 34 homolog); *FZR1* (fizzy/cell division cycle 20 related 1); *CUL3* (cullin 3)	rawP = 5.02e-06;adjP = 2.51e-05	Male-biased
Base excision repair	*HMGB1* (high-mobility group box 1); *POLE3* (DNA-directed polymerase epsilon 3, p17 subunit)	rawP = 0.0004;adjP = 0.0020	Male-biased
Pyruvate metabolism and Fatty Acid Biosynthesis	*ACSS2* (acyl-CoA synthetase short-chain family member 2); *ACACA* (acetyl-Coenzyme A carboxylase alpha)	rawP = 0.0005;adjP = 0.0025	Male-biased
Focal adhesion	*VTN* (vitronectin); *ITGA2* (integrin, alpha 2 subunit of VLA-2 receptor); *COL6A1* (collagen, type VI, alpha 1); *MYLK* (myosin light chain kinase)	rawP = 0.0002;adjP = 0.0030	Female-biased
Fatty Acid Beta Oxidation	*ACADVL* (acyl-Coenzyme A dehydrogenase, very long chain); *LIPC* (lipase, hepatic); *CPT2* (carnitine palmitoyltransferase 2)	rawP = 1.47e-05;adjP = 0.0001	Female-biased
Nuclear receptors in lipid metabolism and toxicity	*ABCC3* (ATP-binding cassette, sub-family C (CFTR/MRP), member 3); *CYP8B1* (cytochrome P450, family 8, subfamily B, polypeptide 1)	rawP = 0.0009;adjP = 0.0063	Female-biased
Metabolism of lipids and lipoproteins	*ABCC3* (ATP-binding cassette, sub-family C (CFTR/MRP), member 3); *ACADVL* (acyl-Coenzyme A dehydrogenase, very long chain); *CPT2* (carnitine palmitoyltransferase 2); *PHYH* (phytanoyl-CoA 2-hydroxylase); *NDUFB8* (NADH dehydrogenase (ubiquinone) 1 beta subcomplex, 8, 19 kDa)	rawP = 2.14e-05;adjP = 0.0012	Female-biased
Integration of energy metabolism	*NP* (nucleoside phosphorylase); *ACADVL* (acyl-Coenzyme A dehydrogenase, very long chain); *CPT2* (carnitine palmitoyltransferase 2); *NDUFB8* (NADH dehydrogenase (ubiquinone) 1 beta subcomplex, 8, 19 kDa); *ACLY* (ATP citrate lyase)	rawP = 2.26e-05;adjP = 0.0013	Female-biased
FOXA transcription factor networks	ACADVL (acyl-Coenzyme A dehydrogenase, very long chain); VTN (vitronectin); UCP2 (uncoupling protein 2 (mitochondrial, proton carrier))	rawP = 0.0001;adjP = 0.0057	Female-biased

Genes mapped to human homologs were submitted to WebGestalt to identify enriched molecular pathway from KEGG database. Hypergeometric test with Bonferroni correction p-value<0.01 were used as statistical filtering criteria. rawP is p-value from hypergeometric test, and adjP is p-value adjusted by Bonferroni multiple test adjustment.

### Common human sex-biased genes and their chromosomal locations

Unlike zebrafish where we only utilized the liver to study toxicological responses, human transcriptomic data were derived from multiple types of tissue and pathological conditions. Thus we first defined common human sex-biased genes based on their sexually dimorphic expression in at least four different tissues out of 9 tissues surveyed. The list of these common sex-biased genes is given in Table 3. There are 7 and 10 common male-biased and female-biased genes, respectively. Most of the common male-biased genes are autosomal genes, except that *PCDH11Y* (protocadherin 11) is located in the Y chromosome and *ASMTL* (O-methyltransferase-like acetylserotonin) in the X chromosome. However, 9 out of 10 common female-biased genes are located in the X chromosome. Interestingly, X-inactive-specific transcript (*XIST*), a long non-coding RNA which is known to play a major role in inactivation of X chromosome [49], [50], was identified as a common female-biased gene. Other X-linked common female-biased genes such as zinc finger proteins *ZFX* and *ZXDA*, together with ribosomal protein S4 (*RPS4X*) and eukaryotic translation initiation factor 1A (*EIF1AX*) that are involved in gene regulation and protein synthesis also appeared as common sex-biased genes in human.

**Table 3:** Common sex-biased expressed genes in human.

Gene Symbol	Gene Name	Associated Sex	Chromosome
*ASMTL*	acetylserotonin O-methyltransferase-like	Male-biased	X
*BIRC6*	baculoviral IAP repeat-containing 6	Male-biased	2
*CS*	citrate synthase	Male-biased	12
*GSTA4*	glutathione S-transferase alpha 4	Male-biased	6
*KLK7*	kallikrein-related peptidase 7	Male-biased	19
*PCDH11Y*	protocadherin 11 Y-linked	Male-biased	Y
*SEMA6A*	sema domain, transmembrane domain (TM), and cytoplasmic domain, (semaphorin) 6A	Male-biased	5
*ARSD*	arylsulfatase D	Female-biased	X
*DDX3X*	DEAD (Asp-Glu-Ala-Asp) box polypeptide 3, X-linked	Female-biased	X
*EIF1AX*	eukaryotic translation initiation factor 1A, X-linked	Female-biased	X
*HOXB2*	homeobox B2	Female-biased	17
*RPS4X*	ribosomal protein S4, X-linked	Female-biased	X
*UTX*	ubiquitously transcribed tetratricopeptide repeat, X chromosome	Female-biased	X
*XIST*	X (inactive)-specific transcript (non-protein coding)	Female-biased	X
*ZFX*	zinc finger protein, X-linked	Female-biased	X
*ZRSR2*	zinc finger (CCCH type), RNA-binding motif and serine/arginine rich 2	Female-biased	X
*ZXDA*	zinc finger, X-linked, duplicated A	Female-biased	X

We defined common sex-biased expressed genes as those genes showing sexual dimorphic expression in at least four different tissue types out of 9 tissues surveyed. Chromosomal location of each gene is obtained from Ensembl Genome Browser.

### Inverted expression of sex-biased genes may be associated with reduced survival fitness

It has been well recognized that sex-biased expressed genes are important for maintenance in sexual reproduction and fitness [51], [52]. Thus, over-expression of sex-biased genes in the opposite sex can be deleterious. This notion is supported by the observation that male fruit flies expressing genes from female-determining genetic loci reduced male fitness [53]. Our analyses provided further evidence that the presence of inverted expression pattern of sex-biased genes in both zebrafish and human are associated to toxico-pathological states. Previous theoretical studies also suggested that sexual selection among males would reduce the equilibrium frequency of deleterious mutations to both sexes [1], [6]. Our analyses revealed that males did generally show greater inverted expression profiles for sex-biased genes under various toxico-pathological states, especially in human pathological cases (Figure 3). Whether these phenomena are truly correlated to evolutionary forces against selection on males for the maintenance of sexual reproduction remained to be investigated.

Currently the exact molecular mechanism leading to observed inverted expression behavior for sex-biased genes under toxico-pathological conditions remains obscure. We believe trans-regulations such as by sex hormones or some sex-biased transcription factors do play the role to certain extent, but their downstream effect may spread to broad network level that is difficult to trace in current study. This also suggests the effect may not come from single axis but multi-layered regulations. However, the discovery of ubiquitous existence of inverted expression profiles for sex-biased genes under broad biological disorders in both fish and human in this study may open a new path to relook how sex-dependent regulation affect cellular functions in near future researches. In summary, our findings of the ubiquity of inverted sex-biased expression profiles under diverse toxico-pathological states suggested the importance of sex-biased genes in normal physiological homeostasis and probably maintenance of sexual reproduction. Both sex-biased genes and their inverted expression profiles are useful for assessing biological disorders and to understand sex differences in pathological incidence, prevalence, and severity.

## Supporting Information

Table S1 Sex-biased expressed genes in the zebrafish liver.
(XLS)
